# The Sequential Action of MIDA9/PP2C.D1, PP2C.D2, and PP2C.D5 Is Necessary to Form and Maintain the Hook After Germination in the Dark

**DOI:** 10.3389/fpls.2021.636098

**Published:** 2021-03-09

**Authors:** Arnau Rovira, Maria Sentandreu, Akira Nagatani, Pablo Leivar, Elena Monte

**Affiliations:** ^1^Plant Development and Signal Transduction Program, Center for Research in Agricultural Genomics (CRAG) CSIC-IRTA-UAB-UB, Barcelona, Spain; ^2^Department of Botany, Graduate School of Science, Kyoto University, Kyoto, Japan; ^3^Laboratory of Biochemistry, Institut Químic de Sarrià, Universitat Ramon Llull, Barcelona, Spain; ^4^Consejo Superior de Investigaciones Científicas (CSIC), Barcelona, Spain

**Keywords:** skotomorphogenesis, etiolation, hook, MIDA9, PP2C.D phosphatases, phytochrome interacting factor PIF, ethylene

## Abstract

During seedling etiolation after germination in the dark, seedlings have closed cotyledons and form an apical hook to protect the meristem as they break through the soil to reach the surface. Once in contact with light, the hook opens and cotyledons are oriented upward and separate. Hook development in the dark after seedling emergence from the seed follows three distinctly timed and sequential phases: formation, maintenance, and eventual opening. We previously identified *MISREGULATED IN DARK9* (*MIDA9*) as a phytochrome interacting factor (PIF)-repressed gene in the dark necessary for hook development during etiolated growth. *MIDA9* encodes the type 2C phosphatase PP2C.D1, and *pp2c-d1/mida9* mutants exhibit open hooks in the dark. Recent evidence has described that PP2C.D1 and other PP2C.D members negatively regulate SMALL AUXIN UP RNA (SAUR)-mediated cell elongation. However, the fundamental question of the timing of PP2C.D1 action (and possibly other members of the PP2C.D family) during hook development remains to be addressed. Here, we show that PP2C.D1 is required immediately after germination to form the hook. *pp2c.d1/mida9* shows reduced cell expansion in the outer layer of the hook and, therefore, does not establish the differential cell growth necessary for hook formation, indicating that PP2C.D1 is necessary to promote cell elongation during this early stage. Additionally, genetic analyses of single and high order mutants in PP2C.D1, PP2C.D2, and PP2C.D5 demonstrate that the three PP2C.Ds act collectively and sequentially during etiolation: whereas PP2C.D1 dominates hook formation, PP2C.D2 is necessary during the maintenance phase, and PP2C.D5 acts to prevent opening during the third phase together with PP2C.D1 and PP2C.D2. Finally, we uncover a possible connection of PP2C.D1 levels with ethylene physiology, which could help optimize hook formation during post-germinative growth in the dark.

## Introduction

When germination takes place in the dark, young seedlings adopt a developmental strategy called skotomorphogenesis or etiolated growth, which is energetically sustained by seed reserves. This dark-growth strategy is characterized by fast hypocotyl elongation to rapidly reach the soil surface, together with the presence of an apical hook and appressed cotyledons, which protect the apical meristem from damage while pushing through the soil (Wei et al., 1994; Gommers and Monte, 2018).

The apical hook structure is a transient structure that develops after germination as a result of the curvature of the hypocotyl apex just below the cotyledons, and protects the apical meristem during emergence from the soil. Hook development proceeds through three different phases: formation, maintenance, and opening (Vandenbussche et al., 2010; Zádníková et al., 2010). The formation phase starts just after germination when the seedling emerges from the seed coat. This phase lasts about 24–36 h in which the hook reaches 180° when completely formed. The maintenance phase follows, in which the hook remains folded for another 24–48 h. Finally, the opening phase starts and hook progressively unfolds to become completely open (angle 0°). The formation phase is achieved by asymmetrical cell expansion and cell division at the apical part of the hypocotyl. Cell expansion is inhibited in the inner (concave) edge of the hook, while cell division and expansion are promoted in the outer (convex) border (Silk and Erickson, 1979; Raz and Koornneef, 2001; Vandenbussche et al., 2010), which forms an apical hook bending of the hypocotyl apex. This asymmetrical cell expansion is caused by an auxin maximum in the concave part of the apical hook. Mutations in auxin transport genes or auxin-synthesis genes cause defects in hook development (Vandenbussche et al., 2010; Zádníková et al., 2010; Willige et al., 2012). In addition to auxin, other hormones like ethylene (ET) and gibberellins (GAs) are involved in hook formation. Exogenous treatment with the ethylene biosynthesis precursor 1-aminocyclo-propane-1-carboxylic acid (ACC) induces a triple response of the etiolated seedlings characterized by an exaggerated hook, short, and thickened hypocotyl. On the other hand, ethylene biosynthetic mutants, as well as ethylene-insensitive mutants, are hookless (Guzmán and Ecker, 1990; Tsuchisaka et al., 2009). GAs also participate in hook formation, given that inactivation of either GAs synthesis or signaling results in a hookless phenotype (Alabadí et al., 2004; Vriezen et al., 2004). Conversely, treatment with GA or mutations in DELLA genes (transcriptional regulators that negatively regulate the GA signaling pathway) exhibit an exaggerated hook (An et al., 2012). It has been proposed that both GAs and ET modulate asymmetrical auxin distribution depending on the apical hook development (Vandenbussche et al., 2010; Zádníková et al., 2010).

The phytochrome-interacting factors (PIFs) are basic helix-loop-helix (bHLH) transcription factors that function as repressors of photomorphogenesis. The PIF quartet (PIFq) members PIF1, PIF3, PIF4, and PIF5 constitutively promote skotomorphogenesis by repressing the photomorphogenesis state in darkness (Leivar et al., 2008b). Single *Arabidopsis thaliana pif1*, *pif3*, *pif4*, and *pif5* mutants show a minor or absent photomorphogenic phenotype in darkness, whereas additive to synergetic effects are observed in higher order mutant combinations. In accordance, *pifq* mutant seedlings display a partial constitutive photomorphogenic phenotype in the dark similar to *cop1* (Leivar et al., 2008b), with short hypocotyl, unfolded hook, and separated cotyledons. Consistent with this observation, the transcriptomic profile of *pifq* mutants in the dark largely resembles that of wild-type (WT) seedlings grown in the light (Leivar et al., 2009; Shin et al., 2009), further illustrating the role of PIFs to maintain the etiolated state. Upon light exposure, PIFs interact with active phytochromes (phy) in their Pfr conformation, and this interaction triggers rapid phosphorylation and proteasome-mediated degradation of PIFs, lifting the repression and allowing photomorphogenesis to initiate (Monte et al., 2004; Al-Sady et al., 2006; Van Buskirk et al., 2012; Leivar and Monte, 2014; Ni et al., 2017). It has been described that cryptochrome (cry) also physically interacts with PIF4 and PIF5 (Pedmale et al., 2016), possibly to repress their transcriptional activity (Ma et al., 2016). Through the action of phys (mainly phyA and phyB) and crys (cry1 and cry2), light rapidly induces complete hook opening, within a few hours (Liscum and Hangarter, 1993; Wu et al., 2010). This hook unfolding occurs due to faster cell elongation at the inner compared to the outer edge (Vandenbussche and Van Der Straeten, 2004; Vriezen et al., 2004). During hook opening, the auxin gradient is greatly reduced (Wu et al., 2010; Willige et al., 2012). Several reports have established direct targets of PIF activity through which PIFs could be contributing to hook development in the dark. PIF5 affects the generation of the auxin gradient by directly regulating *WAG2*, which encodes a protein kinase that regulates auxin transport, and this regulation is modulated by GAs and DELLAs (Willige et al., 2012). PIF5 also directly induce expression of ethylene biosynthesis genes 1-aminocyclopropane-1-carboxulate synthase (ACS) such as ACS5 and ACS8 in a GAs and DELLA-dependent manner (Khanna et al., 2007; Gallego-Bartolomé et al., 2011).

We previously reported a combination of a transcriptomic-based approach with a functional profiling strategy to identify novel regulators of seedling deetiolation downstream of PIF3 (Sentandreu et al., 2011). Four PIF3-regulated genes misexpressed in the dark (*MIDAs*) were defined as novel regulators of seedling deetiolation involved in hypocotyl elongation (*MIDA11*), hook development (*MIDA9* and *MIDA10*), and cotyledon separation (*MIDA1*). Etiolated seedlings deficient in *MIDA9* (*mida9*) showed open hooks after germination and growth in the dark for 4 days (Sentandreu et al., 2011). *MIDA9* encodes the type-2C phosphatase PP2C.D1 belonging to clade D (PP2C.D) of the type-2C phospahatase superfamily in *A. thaliana*. Clade D consists of nine members and is characterized by having a distinct nuclear localization signal and prediction of possible plasma membrane localization (Schweighofer et al., 2004). Recently, MIDA9/PP2C.D1 was reported to modulate the phosphorylation status of the H+-ATPase to regulate cell expansion in the hypocotyl in long day-grown seedlings (Spartz et al., 2014). Interestingly, this activity can be directly inhibited by SMALL AUXIN UP-RNA (SAUR) proteins (Spartz et al., 2014). In addition, expression of *MIDA9/PP2C.D1* has been detected in hypocotyl and hook (Ren et al., 2018). However, the fundamental question of the timing of PP2C.D1 action (and possibly other members of the PP2C.D family) during post-germinative hook development remains to be addressed.

Here, we show that PP2C.D1 is required immediately after germination for hook formation. *mida9/pp2c.d1* shows reduced cell expansion in the outer layer of the hook and, therefore, does not establish the differential cell growth necessary to form the hook, indicating that PP2C.D1 is necessary to promote cell elongation during this early stage. Additionally, genetic analyses of single and high order mutants in PP2C.D1, D2, and D5 demonstrate that the three PP2C.Ds act collectively and sequentially during etiolation. Finally, we uncover a possible connection of PP2C.D1 levels with ethylene physiology, which could help optimize hook formation during post-germinative growth in the dark.

## Materials and Methods

### Plant Material and Seedling Growth

*Arabidopsis thaliana* seeds used here include the previously described *mida9-1* mutant (*pp2c.d1/mida9*) in the ecotype Col-0 background (Sentandreu et al., 2011), and the newly characterized *A. thaliana* lines *pp2c.d2*/SALK_203806 and *pp2c.d5*/SALK_049798 from the Salk Institute Genomic Analysis Laboratory database (Alonso et al., 2003). Homozygous T-DNA insertion lines and WT siblings were identified using PCR with T-DNA and gene-specific primers (Supplementary Table S1). PP2C.D1-YFP transgenic lines were generated by cloning the 2 kb region upstream of the ATG (*PP2C.D1* promoter) in the pDONR-P4-P1R vector, the *PP2C.D1* coding sequence (CDS) in the pDONR-P2R-P3 vector, and the YFP CDS in the pDONR221 vector. LR recombination reaction using the Gateway cloning system (Invitrogen) was done to generate proPP2C.D1::PP2C.D1:YFP in the pH7m34gw vector. The resulting vector was used to transform *pp2c.d1/mida9* to generate PP2C.D1-YFP.

PP2C.D1-GFP-OX transgenic lines were generated by cloning the *PP2C.D1* CDS under the regulation of the 35S promoter in the pH7WG2 vector using the Gateway cloning system. The resulting 35S::PP2C.D1:GFP was used to transform *pp2c.d1/mida9* to generate MIDA9-GFP-OX lines.

Seeds were sterilized and plated on Murashige and Skoog medium (MS) without sucrose as previously described (Monte et al., 2003). For AgNO3 experiments, seeds were sterilized and plated on MS without sucrose with AgNO3 (50 μM). Seedlings were then stratified for 4 days at 4°C in the dark, followed by 3 h of white light to induce germination. Seedlings were then placed in darkness for the indicated period of time. ACC treatments were done as previously described (Gommers et al., 2020) using a concentration of 2 μM.

### Hypocotyl, Hook, and Cell Measurements

For hypocotyl and hook measurements, seedlings grown for 2, 3, and 4 days were arranged horizontally on a plate and photographed using a digital camera (Nikon D80). Measurements were performed using NIH image software (Image J, National Institutes of Health), as described before (Leivar et al., 2008a,b). The angle of a completely closed apical hook was defined as 180°, whereas the angle of a fully opened hook was defined as 0°. Measurements of at least 30 seedlings for each mutant line were tested in R for statically significant differences with the WT sibling controls. Cell size and hook length measurements were visualized at 2-day-old dark-grown seedlings stained with propidium iodine (10 μg/ml; Calbiochem) using a confocal laser microscope Leica SP5 (Emission window: 570–666 nm).

### Gene Expression Analysis

RNA was extracted using the Maxwell RSC plant RNA Kit (Promega). One microgram of total RNA was treated with DNase I (Ambion) according to the manufacturer’s instructions. First-strand cDNA synthesis was performed using the SuperScript III reverse transcriptase kit (Invitrogen) and oligo dT as a primer in the presence of RNase Out (invitrogen). Two microliter of 1:25 diluted cDNA with water was used for real-time PCR (LightCycler 480 Roche) using SYBR Premix Ex Taq (Takara) and primers at 300 nM concentration. *PP2A (AT1G13320)* was used for normalization. Primers used for gene expression analyses are listed in Supplementary Table S2.

### Fluorescence Microscopy

PP2C.D1-YFP and PP2C.D1-GFP were visualized in 2-day-old dark-grown PP2C.D1-YFP and PP2C.D1-GFP-OX seedlings, respectively, using a confocal laser scanning microscope Olympus FV1000 (Emission window: 500–600 nm). *pp2c.d1/mida9* was used as a negative control.

### Protein Extraction and Immunoblots

Protein extracts were prepared from 2-day-old dark-grown seedlings. Tissue samples were collected and frozen in liquid nitrogen. Samples were manually ground under frozen conditions and resuspended in extraction buffer [100 mM MOPS (pH 7.6), 2% SDS, 10% glycerol, 4 mM EDTA, 50 mM sodium metabisulfite (Na_2_S_2_O_5_), 2 μgl^−1^ apoprotein, 3 μgl^−1^ leupeptine, 1 μgl^−1^ pepstatin, and 2 mM PMSF; Al-Sady et al., 2006; Martín et al., 2018].

Total protein was quantified using a Protein DC kit (Bio-Rad), and β-mercaptoethanol was added just before loading. For each sample, 100 μg were treated for 5 min at 95°C and subjected to 12.5% SDS-PAGE gels. Proteins were then transferred to Immobilon-P membrane (Millipore), and immunodetection of PP2C.D1-GFP was performed using an anti-GFP antibody (1:10,000 dilution). Peroxidase-linked anti-rabbit secondary antibody (1:10,000) and SuperSignal West Femto chemiluminescence kit (Pierce) were used for detection of luminescence using a LAS-4000 image imaging system (Fujifilm). The membrane was stained with Ponceau as a loading control.

### Statistical Analysis

Levene’s test was performed to verify equal variances (*p* < 0.05). When the variances were equal, Student *t*-test (*p* < 0.05) or ANOVA test (*p* < 0.05) followed by a *post-hoc* Tukey-b test were performed. For unequal variances, Kruskal-Wallis test (*p* < 0.05) followed by a *post-hoc* Dunn test was performed. To quantitatively assess the magnitude of the difference between ACS time courses, we computed pairwise Euclidean distance (EUC), a well-established method in overall clustering as well as in the calculation of similarity between gene expression time courses (Ramoni et al., 2002). For statistical analyses of ACS time courses, we pooled data for each ACS gene and genotype and used ANOVA test to assess whether the differences across genotypes were significant. All analyses were conducted in R.

## Results

### MIDA9/PP2C.D1 Is Required for Hook Formation During Skotomorphogenic Development

**Figure 1:**
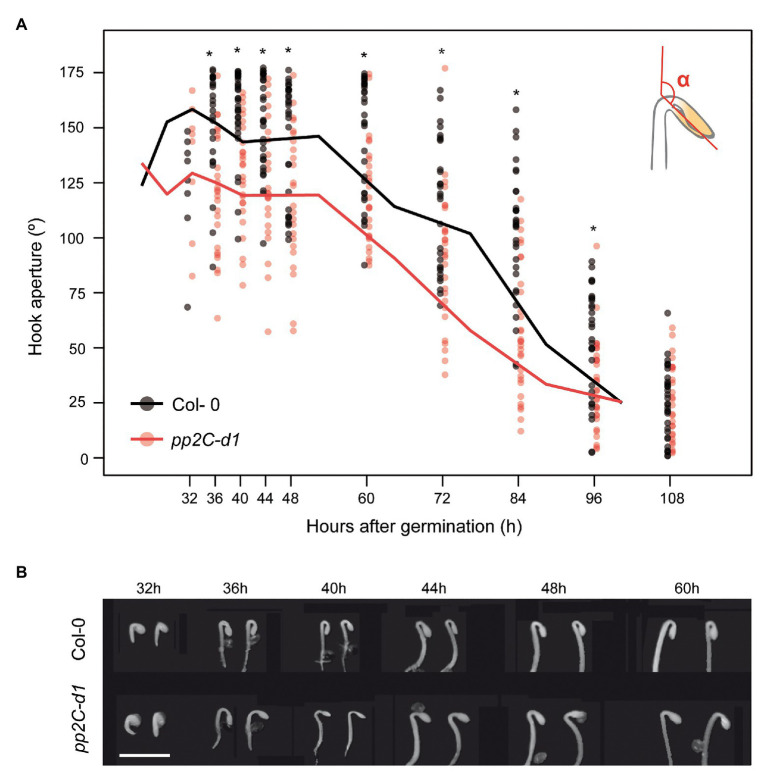
MIDA9/PP2C.D1 is necessary to induce hook formation after germination. **(A)** Time course analysis of apical hook aperture after germination in the dark in Col-0 and *mida9/pp2c-d1*. Lines represent mean values and dots indicate each measurement. Statistical significance relative to Col-0 is indicated by an asterisk (Student *t*-test, *p* < 0.05) *n* = 40. **(B)** Visible phenotypes of seedlings grown in the dark are shown. Bar = 2 mm.

To characterize in detail the role of MIDA9/PP2C.D1 during apical hook development after germination in the dark, we followed the apical hook dynamics in WT (Col-0) and *mida9/d1* mutant seedlings lacking *MIDA9/PP2C.D1* (Sentandreu et al., 2011). The three phases in hook development (formation, maintenance, and aperture) were monitored by measuring the angle of hook curvature at different time points after germination under dark conditions. In Col-0, we observed that under our conditions, hook formation took place after seed emergence during the first 40 h post-germination, reaching a hook closure of 160°. The maintenance phase followed and lasted up to 60 h after germination. Finally, hook opening started (>60 h post-germination) resulting in hook unfolding (about 25°) at 108 h after germination (Figures 1A,B). Compared to Col-0, *pp2c-d1* mutants emerged from the seed coat at the same time. Interestingly, *pp2c-d1* mutants were not able to fully form the apical hook during the formation phase and reached only about 125° of hook closure (Figures 1A,B). These differences between Col-0 and *pp2c-d1* were maintained during the maintenance phase. Finally, hook opening started at similar time in both genotypes and eventually resulted in similar hook unfolding in both genotypes (Figure 1). Together, these data extend our observation that MIDA9/PP2C.D1 participates in hook development as a positive regulator (Sentandreu et al., 2011), and indicate that this regulation occurs specifically during hook formation when PP2C.D1 activity is required in the first hours of post-germinative growth to form the hook.

### MIDA9/PP2C.D1 Is Required to Establish the Asymmetric Growth Necessary for Hook Formation

**Figure 2:**
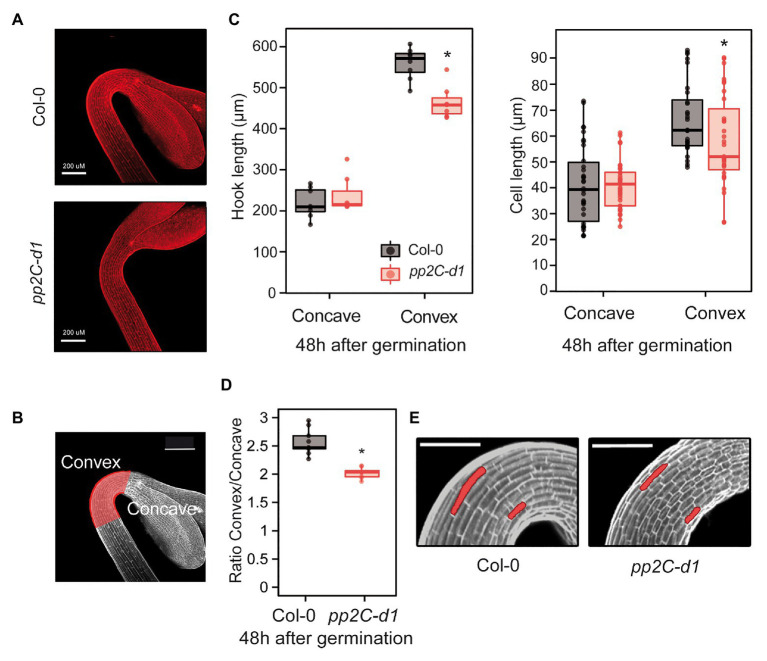
MIDA9/PP2C.D1 induces cell expansion in the outer edge of the apical hook. **(A)** Visual phenotypes of the apical hook in 2-day-old dark-grown Col-0 and *pp2c-d1*. Bar = 200 μm. **(B)** The region of the apical hook is highlighted in red indicating the convex and concave sides. Bar = 200 μm. **(C)** Hook length (left panel) and cell length (right panel) measurements in the concave and convex sides of the apical hook in 2-day-old dark-grown Col-0 and *pp2c-d1*. Dots indicate each measurement. Statistical significance relative to Col-0 is indicated by an asterisk. Right panel (Student *t*-test, *p* > 0.05). Left panel, (Kruskal-Wallis, *p* > 0.05), *n* = 15. **(D)** Apical hook length ratio between convex and concave in Col-0 and *pp2c-d1*. Data are from panel C. Statistical significance relative to Col-0 is indicated by an asterisk (Student *t*-test, *p* < 0.05). **(E)** Visual phenotypes of 2-day-old dark-grown Col-0 and *pp2c-d1*. Cells from concave and convex parts of the hook are highlighted. Bar = 60 μm.

To understand how PP2C.D1 might regulate hook formation, we combined confocal microscopy and phenotypic measurements to study the early stage of *pp2c.d1* hook development in more detail. To examine hook development under dark-grown conditions, we stained Col-0 and *pp2c.d1* with propidium iodide (PI), which is used to visualize plasma membrane delimiting cells (Figure 2A). Because hook formation is achieved mainly as a result of asymmetric elongation of the cells on the outer (convex) edge of the hook compared to the inner edge (concave; Silk and Erickson, 1979; Raz and Koornneef, 2001; illustrated in Figure 2B), we hypothesized that *pp2c.d1* seedlings might be affected in establishing this asymmetric growth. The length of the outer and the inner border of the hook was measured in 2-day-old dark-grown seedlings when the hook formation phase is completed. Whereas the inner concave side was similar in Col-0 and *pp2c.d1*, the outer convex side was significantly longer in Col-0 compared to *pp2c.d1* (Figure 2C left panel). Cell length of the outer and the inner side of the hook was also measured and compared, and no differences in cell length were found in the inner edge of the apical hook in Col-0 and *pp2c.d1*. However, cells in the outer convex side of the hook were longer in Col-0 compared to *pp2c.d1* [Figures 2C (right panel),E]. No apparent difference in cell number was observed between both genotypes. As a result, the convex/concave ratio was higher in Col-0 compared to *pp2c.d1* (Figure 2D), indicating that asymmetric growth in the hook structure in *pp2c.d1* was less pronounced than that of Col-0. Together, these results suggest that PP2C.D1 induces the elongation of the cells in the outer edge of the hook necessary to establish the asymmetric growth that results in the hook organ formation after germination in the dark.

### MIDA9/PP2C.D1 Localization in the Apical Hook

**Figure 3:**
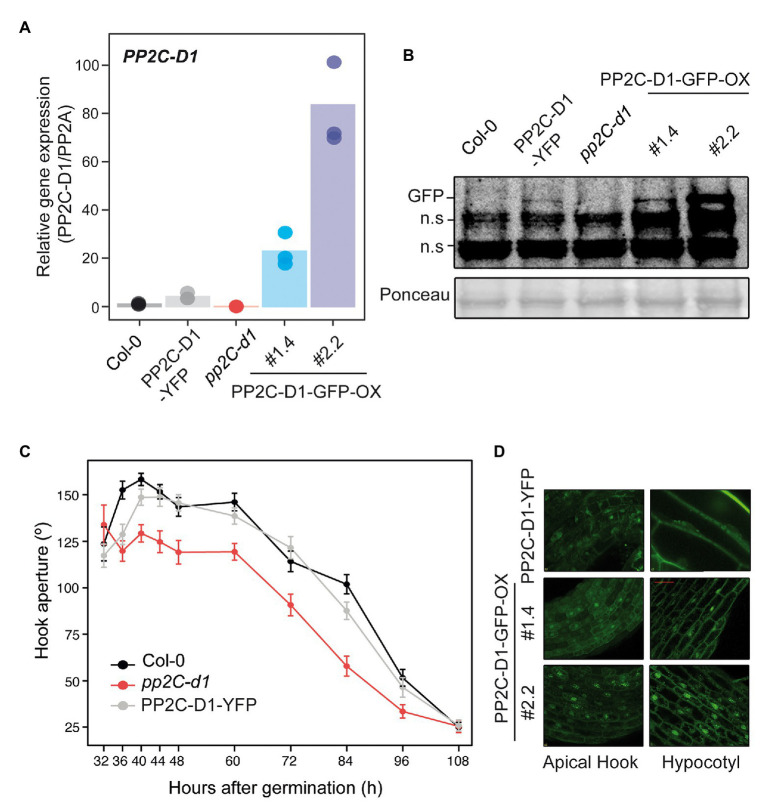
MIDA9/PP2C.D1 is localized to the nucleus and cytoplasm in darkness. **(A)** qRT-PCR analysis of 2-day-old dark-grown Col-0, PP2C.D1-YFP, *pp2c-d1*, PP2C.D1-GFP-OX #1.4, and PP2C.D1-GFP-OX #2.2. *PP2C.D1* expression levels were normalized to *PP2A* and expressed relative to the Col-0 value set at unity. Bars represent mean values and dots indicate each measurement. *n* = 3 biological replicates. **(B)** Immunoblot of protein extracts of 2-day-old dark-grown Col-0, PP2C.D1-YFP, *pp2c-d1*, PP2C.D1-GFP-OX #1.4, and #2.2 seedlings. Protein extracts from Col-0 and *pp2c-d1* were used as negative control. GFP-specific polyclonal antibody was used as a probe. Ponceau staining was used as a loading control. Non-specific cross-reacting bands are marked as n.s. **(C)** Time course analysis of apical hook aperture after germination in the dark of Col-0, *pp2c-d1* and PP2C.D1-YFP. Lines indicate mean values. Error bars indicate s.d. *n* = 40. **(D)** Confocal microscopy images of PP2C.D1-YFP and PP2C.D1-GFP-OX #1.4 and #2.2 in 2-day-old dark-grown seedlings.

Transgenic lines were generated expressing fluorescent-tagged fusions under the strong constitutive 35S promoter (PP2C.D1-GFP-OX) or the endogenous PP2C.D1 promoter (PP2C.D1-YFP) in a *pp2c.d1* mutant background (see Materials and Methods section for details). Compared to Col-0, *PP2C.D1* expression levels were about 4-fold higher in PP2C.D1-YFP lines, and 20- and 80-fold higher in in PP2C.D1-GFP-OX #2.2 and PP2C.D1-GFP-OX #1.4, respectively. We did not detect *PP2C.D1* expression in *pp2c.d1*, consistent with previous results (Sentandreu et al., 2011; Figure 3A). Western blot analyses confirmed higher accumulation of the fusion protein in the overexpressing PP2C.D1-GFP-OX lines with respect to the PP2C.D1-YFP (Figure 3B).

PP2C.D1-YFP transgenic line complemented the hook formation phenotype of *pp2c.d1* (Figure 3C), indicating that the expressed fusion protein is active and functional. Visualization of 2-day-old dark-grown PP2C.D1-YFP by confocal microscopy showed subcellular localization of PP2C.D1 to the nucleus (consistent with the predicted nuclear localization signal; Schweighofer et al., 2004) but also in the cytoplasm, in apical hook and hypocotyl cells (Figure 3D). A comparable pattern, although with increased signal, was observed in cells of the PP2C.D1-GFP-OX lines.

### Sequential Activity of PP2C.D1, D2, and D5 During Hook Formation and Maintenance

We aimed to assess the role of two other members of the PP2C.D clade during the different phases of hook development after germination in the dark. We used single and higher order mutants of PP2C.D1, PP2C.D2 (*AT3G17090*), and PP2C.D5 (mutated in *AT4G38520*). The *pp2c-d1*, *-d2*, and *-d5* mutant alleles used here differ from previous works (Spartz et al., 2014; Ren et al., 2018). All combinations of *pp2c.d1*, *pp2c.d2*, and *pp2c.d5* double and triple mutants were generated, and the three phases in hook development (formation, maintenance, and aperture) were monitored by measuring the angle of hook curvature over 4 days after germination in the dark.

**Figure 4:**
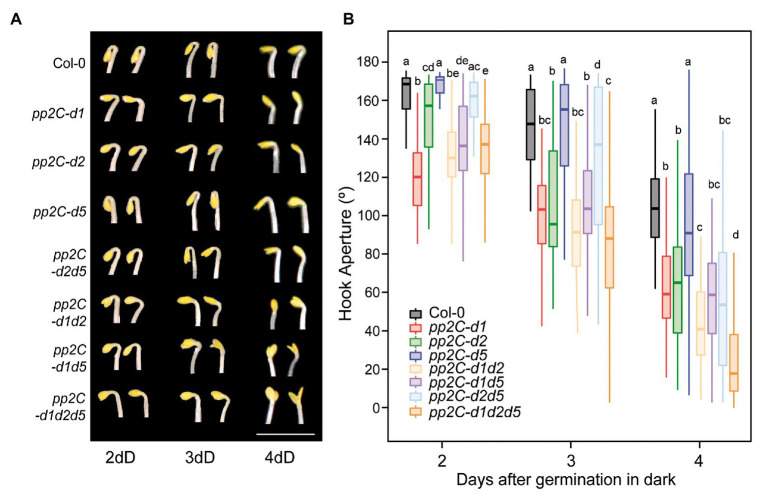
PP2C.D members have distinct temporal functions regulating hook development. **(A)** Visible hook phenotypes of seedlings grown at 2, 3, and 4 days in the dark. Bar = 3 mm. **(B)** Apical hook aperture was measured in 2-, 3-, and 4-day-old dark-grown seedlings in Col-0, *pp2c-d1*, *pp2c-d2*, *pp2c-d5*, *pp2c-d2d5*, *pp2c-d2d1*, *pp2c-d5d1*, and *pp2c-d1d2d5*. Different letters denote statistical differences between means by Kruskal-Wallis test (*p* < 0.05) followed by a *post-hoc* Dunn test. *n* = 40.

Single mutant analysis at 2 days during the formation phase showed a prominent open hook in *pp2c.d1* (Figure 4), consistent with our previous observations (Figure 1). A significant but relatively minor phenotype was observed in *pp2c.d2*, whereas *pp2c.d5* showed no apparent hook phenotype compared to Col-0. Later during the maintenance (3 days) and at the aperture (4 days) phases, *pp2c.d2* mutant displayed a prominent open hook phenotype similar to *pp2c.d1*, and *pp2c.d5* did not show an observable phenotype. Together, these results suggest that (i) PP2C.D1 and PP2C.D2 act together to promote hook formation, with a dominant role of PP2C.D1 and a relatively marginal role of PP2C.D2; (ii) PP2C.D2 promotes hook maintenance together with PP2C.D1; and (iii) PP2C.D5 might have a minor or no contribution in the presence of the other clade D PP2C members.

In order to test for possible redundancy between family members, we next characterized higher order mutants between *PP2C.D1*, *PP2C.D2*, and *PP2C.D5*. No significant additive or synergistic genetic interactions were identified at the hook formation phase at 2 days, as the observed differences were relatively minor in magnitude (Figure 4). A similar tendency was observed at the maintenance phase at 3 days, only that the role of PP2C.D2 in maintaining the hook was also evident in the absence of the other two members PP2C.D1 and PP2C.D5 (compare *pp2c.d1d5* with *pp2c.d1d2d5*). Interestingly, additive/synergistic genetic interactions were clearly observed at the opening phase at 4 days. First, relative to the *pp2c.d1* and *pp2c.d2* single mutants, *pp2c.d1d2* showed a significantly more open hook phenotype. Second, in contrast to 2 and 3 days, a clear contribution of *PP2C.D5* was observed in the triple mutant *pp2c.d1d2d5* in the absence of *PP2C.D1* and *PP2C.D2* (compare *pp2c.d1d2* with *pp2c.d1d2d5*).

Together, this genetic analysis unveils a complex scenario where PP2C.D1, PP2C.D2, and PP2C.D5 collectively participate in hook development, but that they do so in a temporal and hierarchical manner. PP2C.D1 has a prevalent role in promoting hook formation, whereas PP2C.D2 plays a relatively marginal role at this phase. In contrast, at the maintenance phase, PP2C.D2 gains quantitative importance. Finally, PP2C.D5, with no apparent role during hook formation or maintenance, acts at the aperture phase to prevent hook opening in combination with PP2C.D1 and PP2C.D2.

### Interplay Between MIDA9 and Ethylene Biosynthesis in the Dark

**Figure 5:**
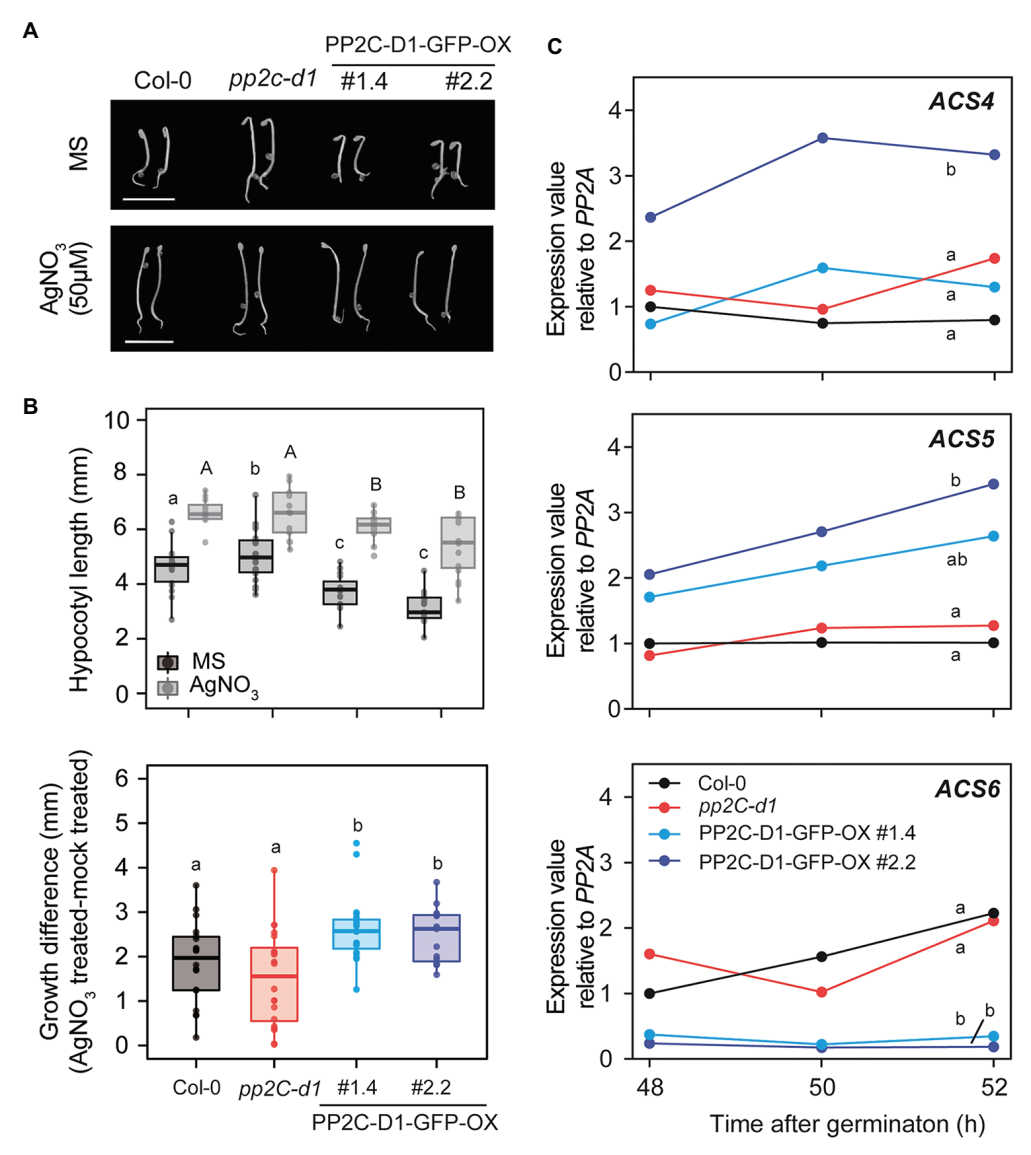
MIDA9/PP2C.D1 participates in ethylene responses modulating 1-aminocyclopropane-1-carboxulate synthase (*ACS*) expression in dark-grown seedlings. **(A)** Visible phenotypes of 2-day-old dark-grown seedlings in MS or MS+ AgNO3 (50 μM) of Col-0, *pp2c-d1*, and PP2C.D1-GFP-OX #1.4 and #2.2. Bar = 5 mm **(B)** Upper panel, hypocotyl length of 2-day-old dark-grown seedlings in MS or MS+ AgNO3 (50 μM) of Col-0, *pp2c-d1*, and PP2C.D1-GFP-OX #1.4 and #2.2. Lower panel, hypocotyl growth differences of AgNO3 (50 μM) treated compared to mock treated plants. **(C)** qRT-PCR time course analysis of Col-0, *pp2c-d1*, and PP2C.D1-GFP-OX #1.4 and #2.2. *ACS* expression levels were normalized to *PP2A* and expressed relative to the Col-0 at 48 h after germination value set at unity. One of two biological replicates with similar results is represented. In panels b and c, different letters denote statistical differences between means by ANOVA (*p* < 0.05) followed by *post-hoc* Tukey-b test.

PP2C.D1-GFP-OX seedlings displayed short and thickened hypocotyls under dark-grown conditions compared to Col-0 and *pp2c.d1* seedlings (Figures 5A,B). This feature has been previously described in mutants with increased ethylene levels (Guzmán and Ecker, 1990), and led us to hypothesize that the observed phenotype of etiolated PP2C.D1-GFP-OX lines may be at least in part related to altered ethylene biosynthesis or response. Treatment with AgNO3 that blocks accessibility to ethylene receptors (Beyer, 1976), resulted in greater hypocotyl elongation responses in PP2C.D1-GFP-OX lines compared to Col-0 in 3-day-old dark-grown seedlings (Figures 5A,B). Hypocotyl growth differences were calculated by subtracting the hypocotyl length of seedlings grown in MS (mock) from those of seedlings grown in AgNO3, and results showed greater difference in MIDA9-GFP-OX compared to Col-0 (Figure 5B lower panel). These results suggest that the short hypocotyl phenotype of MIDA9-GFP-OX lines might be caused by increased ethylene levels, and indicates that the activity of PP2C might impact ethylene biosynthesis or action.

Stimulation of ethylene production is achieved through upregulation of the transcript levels of enzymes involved in ethylene biosynthesis. Conversion of ACC by ACS is the first committed step in ethylene biosynthesis and is considered to be the rate-limiting step. *ACS* is encoded by a multigene family containing at least eight functional members in *A. thaliana* (Yamagami et al., 2003). We analyzed the levels of *ACS4*, *5*, and *6* at 48, 50, and 52 h after germination in the dark, when the short hypocotyl phenotype is robust (Figure 5A). Compared to Col-0, we detected elevated levels of *ACS5* in the PP2C.D1-GFP-OX lines (Figure 5C). In contrast, only PP2C.D1-GFP-OX #2.2 line showed elevated *ACS4* expression levels compared to Col-0. The fold-change increase was greater in the PP2C.D1-GFP-OX #2.2 compared to #1.4, in agreement to #2.2 having greater PP2C.D1 levels compared to #1.4 (Figures 3A,B). In contrast, the *ACS6* expression levels, which are under negative feedback regulation (Chang et al., 2008), were greatly reduced in PP2C.D1-GFP-OX lines compared to Col-0 and *pp2c.d1*. ACS levels were not significantly affected in the *pp2c.d1* mutant. Analysis using a combination of Euclidean distance and ANOVA quantified the differences between time courses and assessed their statistical significance. Col-0 was found to differ the most from PP2C-D1-GFP-OX#2.2 across all three genes in a significant manner (Euclidean distance ACS4: 3.3, ACS5: 2.6, ACS6: 2.6), while Col-0 and *pp2C-d1* were found to be highly similar across all genes (Euclidean distance ACS4: 0.8, ACS5: 0.4, ACS6: 0.8; Figure 5C). PP2C-D1-GFP-OX#1.4 was found to be largely different from Col-0 in ACS5 and ACS6 (Euclidean distance 1.7 and 2.4, respectively), but not in ACS4 (Euclidean distance 0.8). These differences were found to be statistically significant for ACS6 but not ACS4, whereas for ACS5, PP2C-D1-GFP-OX#1.4 was found to be undistinguishable to both WT and PP2C-D1-GFP-OX#2.2 (Figure 5C). Together, these results suggest that PP2C.D1 levels might impact the regulation of ethylene biosynthesis. Interestingly, ACC treatment induced an exaggerated hook in all PP2C.D mutant lines similar to the control (Supplementary Figure S1), suggesting that PP2C.D levels do not affect ethylene signaling mediating hook development.

## Discussion

Although much progress has been achieved in the past years, how PIFs impose the different aspects of deetiolation during seedling establishment still remains incomplete. Our laboratory previously identified the PIF3-regulated *MIDA9* encoding the type-2C phosphatase PP2C.D1 as a gene involved in hook development (Sentandreu et al., 2011). Here, we performed a detailed genetic and phenotypic characterization of the role of *MIDA9/PP2C.D1* and two closely related PP2Cs to define their timing of action in the regulation of the different phases of hook development after germination in the dark (hook formation, maintenance, and opening). We found that MIDA9/PP2C.D1 is required for hook formation and that this is achieved by specifically promoting the elongation of the outer part of the apical hook. Furthermore, we identified sequential activity of PP2C.D1, PP2C.D2, and PP2C.D5 in the three phases of hook development, with some redundant roles. Finally, we described a potential connection between MIDA9/PP2C.D1 and ethylene physiology.

### MIDA9/PP2C.D1 Is Required for Cell Expansion in the Outer Part of the Apical Hook

Apical hook development is a complex process that takes place in three different phases: formation, maintenance, and opening (Vandenbussche et al., 2010; Zádníková et al., 2010). It is, therefore, important to analyze the temporal stages of the complete dynamic process of hook development to understand how it is achieved, as an unfolded hook in mutated etiolated seedlings could be the result of a deficiency in hook formation, a defective maintenance, or a faster opening phase. Our finding that MIDA9/PP2C.D1 is predominantly involved in the first stage of hook formation (Figure 1) suggested that PIF-PP2C.D1 participates in establishing the asymmetric growth that allows bending of the upper part of the hypocotyl to form the hook. Indeed, whereas hook and cell length in the inner part was indistinguishable in WT and *mida9/pp2c.d1* mutants, the mutants did not elongate the cells in the outer convex part of the hook region as much as the WT, resulting in incomplete bending and deficient hook formation (Figure 2). These results indicate that PP2C.D1 functions to induce cell expansion in the outer cell layer of the hook to promote hook formation. This was somewhat unexpected because previous reports proposed that PP2C.D1 inhibits cell elongation, although this was based on hypocotyl data (Spartz et al., 2014). Indeed, PP2C.D1 function to induce cell expansion in the hook is in contrast with the role of PP2C.D1 in the hypocotyl: while *mida9/pp2c.d1* mutants were slightly longer than WT, PP2C.D1 overexpressing plants display shorter hypocotyls (Figure 5; Spartz et al., 2014). These results suggest that PP2C.D1 inhibits cell elongation in the hypocotyl and are in accordance with previous studies in knockdown amiD2/5/7/8/9 seedlings of five other PP2C.D members (Spartz et al., 2014), and with a triple mutant deficient in PP2C.D2, D5, and D6 (Ren et al., 2018), which showed long hypocotyl phenotype and increased cell expansion compared to WT seedlings. Considering these observations, it was concluded that these PP2C.Ds are negative regulators of hypocotyl cell expansion. A described mechanism involves interaction of PP2C.D1 with plasma membrane H+-ATPases to regulate cell hypocotyl expansion (Spartz et al., 2014; Ren et al., 2018), in a process where the auxin-induced SAURs (Small Auxin-Upregulated RNA) interact with PP2C.Ds proteins to inhibit PP2C activity, and allow activity of the H+-ATPase to promote cell expansion. These data, together with our new findings, indicate that MIDA9/PP2C.D1 might play different roles in different organs which could involve interaction with different partners.

A recent study defined localization patterns of PP2C.D1 in nuclei and cytoplasm similar to our results (Ren et al., 2018). However, this study reported stronger localization of PP2C.D1 only in the inner side of the hook, in contrast to our findings (Figure 3D). Intrigued by this, we compared the PP2C.D1 promoter used in each construct. Significantly, Ren et al. (2018) used 4.4 kb upstream of the transcriptional start site (TSS) of PP2C.D1, whereas here we used a shorter promoter of 2 kb. This suggests that the region between 2 and 4.4 kb upstream of the TSS might contain regulatory elements required for the asymmetric expression in the hook. Interestingly, our results suggest that PP2C.D1 function does not seem to require asymmetric localization, as PP2C.D1-YFP can complement the hook formation phenotype of *pp2c.d1* (Figure 3C). A possible alternative explanation is asymmetrical distribution of a necessary partner. Further analyses will be necessary to determine how PP2C.D1 localization is correlated with its function.

### A Connection Between MIDA9/PP2C.D1 and Ethylene Physiology in Etiolated Seedlings

It has been described that hook formation takes place by asymmetric growth of the top part of the hypocotyl as a result of inhibition of cell expansion at the inner side of the hook coinciding with a local maxima of the auxin gradient (Silk and Erikson, 1979; Raz and Koornneef, 2001). However, our results showing that *pp2c.d1* has impaired cell elongation in the outer cells of the hook (Figure 2) suggest that cell elongation in the outer side is also highly regulated, and that this process requires PP2C.D1. Interestingly, the recent results showing asymmetric accumulation of PP2C.D1 in the hook (Ren et al., 2018) are reminiscent of the asymmetric accumulation of auxin during hook formation, suggesting that PP2C.D1 might be involved in the hormonal regulation of this process. In fact, several PP2Cs have been long-known to be related to hormone responses. The first PP2C in plants was identified as a mutation that gave rise to an ABA-insensitive phenotype of the mutant *abi1* (ABA insensitive 1; Meyer et al., 1994). Moreover, the homologous of ABI1, ABI2 (ABA insensitive 2), was also found to mediate the full range of ABA responses (Rodriguez et al., 1998). These results were the first evidence that PP2C proteins are connected with phytohormones to mediate stress signaling responses. Furthermore, one member of clade F (*PIA1*) also appears to be involved in hormone-mediated responses, by regulating the accumulation of stress hormones such as ethylene and salicylic acid (Widjaja et al., 2010).

Our finding that overexpressing PP2C.D1-GFP lines have altered expression of genes involved in ethylene biosynthesis (*ACS*) in etiolated seedlings suggest that PP2C.D1 might regulate hook formation through affecting ethylene accumulation. Different studies have demonstrated that ethylene is involved in the regulation of hook development, with a role during the formation and maintenance phase of hook development through the induction of auxin biosynthesis and regulating its transport and signaling in the apical hook (Raz and Koornneef, 2001; Mazzella et al., 2014). In fact, dark-grown seedlings treated with the ethylene biosynthesis precursor ACC exhibit an exaggerated apical hook, which is one of the components of the classical triple response together with shortening and thickening of both the hypocotyl and root and a proliferation of root hairs. Interestingly, whereas the levels of overexpression of PP2C.D1 in PP2C.D1-GFP were enough to induce a short hypocotyl, they did not promote exaggerated apical hook, suggesting that necessary partners for PP2C.D1 function in hook formation might be limitant, or that the levels of PP2C.D1 in our overexpressing lines were not enough to elicit a hook phenotype. In favor of these latter possibility, a recent paper by Wang et al. (2020) reports PP2C.D1 overexpressor lines that have a more closed apical hook in the dark. Although our data do not allow us to conclude that PP2C.D1 function in hook formation involves regulation of ethylene biosynthesis, our results suggest a possible connection between PP2C.D1 activity and ethylene physiology that has not been previously recognized. This connection does not seem to involve ethylene signaling, as ACC treatment promoted an exaggerated hook in all our mutant lines. Further experiments should be performed to elucidate the nature of the connection between PP2C.D1 and ethylene.

### Hierarchical Role of PP2C.Ds in the Different Stages of Hook Development

Our temporal analysis of single, double, and triple mutants of the PP2C.D family suggest that they participate in a hierarchical and complex manner during hook development. We defined that PP2C.D1 has a predominant role in promoting hook formation and maintenance, while PP2C.D2 showed a negligible role during hook formation but contributes to maintenance similarly to PP2C.D1, and PP2C.D5 acts in the aperture phase to prevent early hook opening in combination with PP2C.D1 and PP2C.D2. Some of these functions became more apparent in high order mutants, suggesting a degree of redundancy among PP2C.D members in the regulation of hook development. Redundancy in hook development has been also shown in the triple mutant *pp2c-d2/d5/d6*, although authors did not explore in the distinct hook development phases (Ren et al., 2018).

The temporal sequential role of PP2C.D1, PP2C.D2, and PP2C.D5 in hook development and the dominant function of PP2C.D1 might be determined by differential abundance, location pattern, and/or activity of these members along the process. It is also possible that the different stages require a different protein accumulation threshold. Ren et al. (2018) examined the expression patterns of *PP2C.D* genes by using *pPP2C.D-GUS* reporter fusions in 3-day-old etiolated seedlings, and found that, whereas PP2C.D1 is almost exclusively expressed in the hook area, PP2C.D2 and PP2C.D5 are distributed more broadly and display high levels in the hypocotyl and low levels in the hook area. This pattern suggests that the dominant function of PP2C.D1 during hook development compared to other PP2C.Ds might be due to its prominent expression in the hook region among the PP2C.D family. Analyses of the accumulation kinetics of PP2C.D1, PP2C.D2, and PP2C.D5 during the different phases of hook development would be necessary to assess whether their temporal accumulation pattern correlate with their sequential function.

## Data Availability Statement

The original contributions presented in the study are included in the article/Supplementary Material, further inquiries can be directed to the corresponding author.

## Author Contributions

All authors contributed to design the work. AR and MS acquired and analyzed data. AR, PL, and EM wrote the manuscript. All authors contributed to the article and approved the submitted version.

### Conflict of Interest

The authors declare that the research was conducted in the absence of any commercial or financial relationships that could be construed as a potential conflict of interest.
