# Supplementary material for: The Sequential Action of MIDA9/PP2C.D1, PP2C.D2, and PP2C.D5 Is Necessary to Form and Maintain the Hook After Germination in the Dark
Source: Front Plant Sci. 2021 Mar 9;12:636098. doi: 10.3389/fpls.2021.636098 (PMC7985339; doi:10.3389/fpls.2021.636098)
Supplement: Supplementary file 1 [file Data_Sheet_1.pdf]

SUPPLEMENTAL TABLES

Table S1. Primers used to genotype new T-DNA mutant lines.

Mutant line	AGI	PCR Reaction	Forward	Reverse
pp2c-d2	AT3G17090	WT	GGTGATGAAGATGGTGATTGG	TCATCAGAAACCCATTACTTGC
		T-DNA	TGGTTCACGTAGTGGGCCATC	TCATCAGAAACCCATTACTTGC
pp2c-d5	AT4G38520	WT	GAGAAGTCACCAAAGACGTGC	ATTCTTTGCCACTGTTTGGTG
		T-DNA	TGGTTCACGTAGTGGGCCATC	ATTCTTTGCCACTGTTTGGTG

Table S2. Primers used for gene expression analyses.

Gene	AGI	Reference	Forward	Reverse
ACS4	AT2G22810	Thain *et al.*, 2004	GTTTACGAAGTGAAGCTCAAC	GTCTCATCAATCATGTTCGCG
ACS5	AT5G65800		GCGGCAAGTCTCAAGAGGA	TTCTGGGCTTGTTGGTAAGC
ACS6	AT4G11280		GTTCCAACCCCTTATTATCC	CCGTAATCTTGAACCCATTA
PP2A	AT1G13320	Shin *et al.*, 2007	TATCGGATGACGATTCTTCGT	GCTTGGTCGACTATCGGAATG

References

Thain, S.C., Vandenbussche, F., Laarhoven, L.J., Dowson-Day, M.J., Wang, Z.Y., Tobin, E.M., Harren, F.J., Millar, A.J., Van Der Straeten, D. (2004). Circadian rhythms of ethylene emission in Arabidopsis. *Plant Physiol.* 136, 3751-3761.

Shin, J., Park, E., Choi, G. (2007). PIF3 regulates anthocyanin biosynthesis in an HY5-dependent manner with both factors directly binding anthocyanin biosynthetic gene promoters in Arabidopsis. *Plant J.* 49, 981–994.
