# Supplementary material for: The Sequential Action of MIDA9/PP2C.D1, PP2C.D2, and PP2C.D5 Is Necessary to Form and Maintain the Hook After Germination in the Dark
Source: Front Plant Sci. 2021 Mar 9;12:636098. doi: 10.3389/fpls.2021.636098 (PMC7985339; doi:10.3389/fpls.2021.636098)
Supplement: Supplementary file 2 [file Data_Sheet_2.pdf]

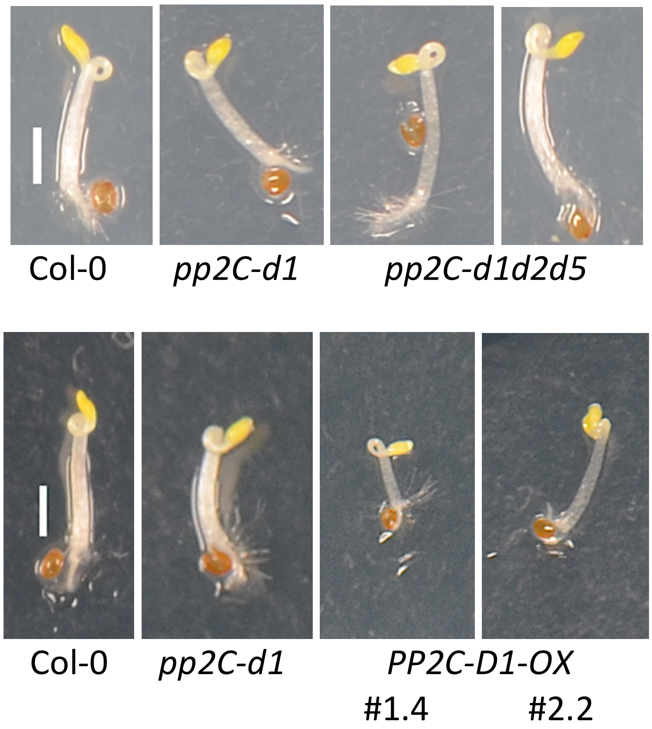


**Supplementary Figure S1. ACC-induced hook curvature in PP2C.D mutants.** Visual phenotypes of 3-day-old (Top) and 4-day-old (Bottom) etiolated Col-0, *pp2c-d1*, *pp2c-d1d2d5* and *PP2C-D1-OX* seedlings grown in the dark on MS medium containing 2 µM ACC. Bar = 1 mm.
